# Dual-source CT angiography of the thoracic aorta using prospective cardiac gating and a low kilovoltage technique

**DOI:** 10.1186/1532-429X-11-S1-P251

**Published:** 2009-01-28

**Authors:** Cormac Farrelly, Aoife Keeling, John Sheehan, Vahid Yaghmai, James Carr

**Affiliations:** Northwestern Memorial Hospital, Chicago, IL USA

**Keywords:** Thoracic Aorta, Image Noise, Point Spread Function, Effective Radiation Dose, Cardiac Gating

## Introduction

Cardiac gating leads to less artifacts and more accurate measurements of the thoracic aorta and root. Without gating the aortic root and ascending aorta are particularly prone to artifact. To date gating has lead to increased dose to patients who often have to undergo multiple studies.

## Purpose

To investigate the effects of prospective cardiac gating and low kilovoltage parameters on image quality and radiation dose when acquiring CT angiography of the thoracic aorta (CTTA).

## Methods

Dual-source CTTA was performed on 60 consecutive patients. One group of thirty were examined with retrospective gating and standard parameters (120 kV, 340 mAs). The other thity were examined with prospective gating and low dosage parameters (100 kV, 170 mAs) with the scanner in a Step and Shoot (SAS) mode that automatically recognizes and does not acquire data during ectopic heart beats. Qualitative analysis was performed by two blinded reviewers who assessed image quality and graded it on a three point scale. Sharpness of the thoracic aorta was also evaluated quantitatively at three levels by generating a line profile across the aortic vessel wall and calculating the point spread function. Both sides of the density profile were analyzed, averaged and then used to calculate sharpness (Hounsfield units/mm). Attenuation in the aorta and image noise was measured. Radiation dose was measured.

## Results

Qualitative and quantitative analysis of sharpness of the thoracic aorta and image noise showed no significance difference (p > 0.05) between the two patient groups. Estimated effective radiation dose of the prospective low kilovoltage protocol (mean dose <3 mSv) was significantly lower (p < 0.01).

## Conclusion

Low dose imaging of the thoracic aorta with maintenance of image quality and sharpness is achievable using a prospective cardiac gated low kilovoltage technique.

